# Cross-Species Extrapolation of Prediction Model for Lead Transfer from Soil to Corn Grain under Stress of Exogenous Lead

**DOI:** 10.1371/journal.pone.0085688

**Published:** 2014-01-08

**Authors:** Zhaojun Li, Hua Yang, Yupeng Li, Jian Long, Yongchao Liang

**Affiliations:** 1 Institute of Agricultural Resources and Regional Planning, Chinese Academy of Agricultural Sciences, Key Laboratory of Plant Nutrition and Fertilizer, Ministry of Agriculture, Beijing, China; 2 Guizhou Key Laboratory of Mountain Environment, Guizhou Normal University, Guiyang, China; 3 College of City and Environmental Sciences, Shanxi Normal University, Linfen, China; University of Kentucky, United States of America

## Abstract

There has been increasing concern in recent years regarding lead (Pb) transfer in the soil-plant system. In this study the transfer of Pb (exogenous salts) was investigated from a wide range of Chinese soils to corn grain (Zhengdan 958). Prediction models were developed with combination of the Pb bioconcentration factor (BCF) of Zhengdan 958, and soil pH, organic matter (OM) content, and cation exchange capacity (CEC) through multiple stepwise regressions. Moreover, these prediction models from Zhengdan 958 were applied to other non-model corn species through cross-species extrapolation approach. The results showed that the soil pH and OM were the major factors that controlled Pb transfer from soil to corn grain. The lower pH and OM could improve the bioaccumulation of Pb in corn grain. No significant differences were found between two prediction models derived from the different exogenous Pb contents. When the prediction models were applied to other non-model corn species, the ratio ranges between the predicted BCF values and the measured BCF values were within an interval of 2-fold and close to the solid line of 1∶1 relationship. Moreover, the prediction model i.e. Log[BCF] = −0.098 pH-0.150 log[OM] −1.894 at the treatment of high Pb can effectively reduce the measured BCF intra-species variability for all non-model corn species. These suggested that this prediction model derived from the high Pb content was more adaptable to be applied to other non-model corn species to predict the Pb bioconcentration in corn grain and assess the ecological risk of Pb in different agricultural soils.

## Introduction

Lead (Pb) is one of the most toxic heavy metals for people in the world [1]–[3]. In children, Pb has been known to cause decreases in intelligence quotient (IQ) scores, retardation of physical growth, and hearing problems. In individuals of all ages, it has been known to cause anemia brain damage, impaired function of the peripheral nervous system, high blood pressure, reproductive abnormalities, development defects, abnormal vitamin D metabolism, and in some situations death of people [4]–[5]. Soil-to-plant transfer of heavy metals is the major exposure pathway for humans with regards to soil contamination [6]. Therefore, the accumulation of heavy metals in plants in agricultural soils, especially in edible part of plants, is great concern regarding its posing potential threats [7]–[8]. The efficiency of the plants for absorbing metals is mainly evaluated by plant uptake or the metal transfer factors from soil to plant [9]. Evaluating the metal transfer factors from soil to plant especially to edible parts can further provide the information about accumulation of contaminants into the food chain [10].

The Pb transfer factor from soil to plant mainly depends on its availability in soils. Soil properties can determine the availability of Pb to crops, including soil pH, organic matter (OM), cation exchange capacity (CEC), redox potential, clay minerals, Fe and Mn oxides, and calcium carbonate content [11]–[13]. In addition, it has been reported that soil pH is a key factor in controlling the cation mobility and regulating the solubility of heavy metals in soils [14]–[15]. The heavy metals tended to be more available in acid soil [16]. The low soil pH is favorable for the transference of Pb from soil to plant [17]–[19]. As well as, soil OM has also been found to play an important role in influence Pb adsorption processes in soils [20]–[21]. Pb in soils with low OM content was difficult to be adsorbed but was easy to be uptaken by plants [22]. The importance for assessing the ecological risk of heavy metals in complex soil types has urged a lot of prediction models to be developed to predict heavy metals transfer from soil to plant [23]. The heavy metal transfer factor from soil to plant also depends on plant species. It has been observed that significant differences exist in heavy metal uptake among cultivars [24]. Due to the expenses and efforts involved, these models were limited to specific contaminated sites or single species [25]–[27]. In addition, most of these models were developed to describe the transfer of heavy metals from soil to plant leaves, vegetables, earthworms by using the log-transformed concentrations [28]–[29]. A few attempts were only made to describe heavy metals uptake by rice, wheat and barley grain directly from soil with combination of total concentrations of heavy metal and soil basic properties [30]–[31]. Little is reported about the prediction models developed to describe the transfer of heavy metals, especially Pb, from soils to corn grain. Intake of heavy metals from dietary sources may represent a significant exposure pathway for human populations. Prediction of heavy metals including Pb concentrations in edible part of crops based on their concentration in soil and other environmental factors are urgently required for human risk assessment and derivation of soil environmental benchmark croplands.

The species sensitivity distributions (SSD) have been proved to be successful and were increasingly used in ecological risk assessment procedures [32]–[33]. However, the toxicology data from the soils with different soil properties need to be normalized using prediction models to eliminate the influence of soil factors [34]. It can improve the accuracy of the sensitivity distribution of species and environmental quality standard values [35]. These models can also provide great opportunities to carrying out ecological risk assessments and establishing soil quality criteria for heavy metals in croplands. Due to the fact that significant differences existed among cultivars for heavy metal uptake, it is ideal that separate species-specific models should be developed for each single species. However, this is unrealistic due to the expenses and efforts involved. Thus the cross-species extrapolation of toxicity data models was often applied in some cases of pollutants risk assessment practice. For example, Deleebeeck et al. [36]–[37] have applied biotic bigand model (BLM) from *O. mykiss* to other fish species and applied BLM from D. *magna* to other cladocera species. Schlekat et al. [38] applied chronic nickel BLMs developed for the cladocera such as Daphnia magna and Ceriodaphnia dubia to predict chronic toxicity of nickel to three other invertebrates including snail *(Lymnaea stagnalis*), insect (*Chironomus tentans*), and rotifer (*Brachionus calyciflorus*). Yang et al. [16] applied corn grain Cd BCF predication models developed for Zhengdan 958 to predict other non-model corn species and wheat grain Cd BCF. However, little effort has been put on establishing models to describe the relationship between Pb accumulation in corn grain and soil properties. The objectives of this study are: 1) to establish the prediction model for Pb accumulation in corn grain on seventeen soils with greatly different properties; 2) to assess the feasibility of applying these models to other non-model corn species, and 3) to investigate their accuracy in predicting accumulation of Pb in non-model corn species.

## Materials and Methods

None of these 17 soil samples were collected from national parks or other protected areas. It is confirmed that no specific permissions were required for the soils sampling activities in the 17 locations in China. It is also can be confirmed that the field studies did not involve endangered or protected species. No tested corn species are under first- or second-class state protection, and they are not listed in the Inventory of Rare and Endangered Plants of China (http://zrbhq.forestry.gov.cn/portal/zrbh/s/3053/content-457748.html), or the Key Protected Inventory of Wild Plants of China (http://zrbhq.forestry.gov.cn/uploadfile/zrbh/2010-10/file/2010-10-14-bb296addeaa047798d6b6c476aaa1da9.doc). These corn species were used for only scientific research as permitted by the Ministry of Agriculture of China.

### Soil Samples

A set of 17 soils covering a wide range of soil properties was sampled from typical locations in China. In each sample location, the soils were collected from the top 20 cm of the soil profile. For analysis of their phys-chemical characteristics, the soils were air dried and sorted to pass a 2-mm sieve. Soil pH was measured in deionized water (soil: solution ratio, 1∶5) [39]. CEC was determined by the unbuffered silver-thiourea method [40]. OM was measured by dry combustion [41]. The total phosphorus in soils (TP) was measured by colorimetric method [42]. The total nitrogen in soils was determined by the Kjeldahl’s method [43]. The background Pb content in the soils was determined by aqua regia (1∶3 fresh mixture of concentrated HNO_3_ and HCl) digestion [44]. The selected properties of the 17 soil samples were shown in Table 1.

**Table 1:** Selected soil properties of the 17 soils used in the lead bioconcentration factor test.

Soil NO.	Location*	pH	OM (g.kg^−1^)	CEC (cmol.kg^−1^)	TN (g.kg^−1^)	TP (mg.kg^−1^)	TK (g.kg^−1^)	Background Pb (mg.kg^−1^)
S1	Hunan	4.90	15.52	10.85	1.14	0.47	15.26	23.12
S2	Chongqing	5.74	17.48	21.34	1.00	0.55	22.61	29.72
S3	Liaoning	5.74	25.84	12.19	1.00	0.73	23.94	27.94
S4	Yunnan	5.92	34.26	11.10	2.01	0.81	4.77	24.58
S5	Jiangxi	6.01	11.69	8.70	0.51	0.52	9.96	33.48
S6	Anhui	6.25	20.04	19.08	0.99	0.35	15.41	29.34
S7	Heilongjiang	6.27	35.69	28.59	1.74	0.48	24.70	33.99
S8	Jilin	6.82	32.85	31.11	1.75	0.35	24.58	31.01
S9	Jiangsu	6.93	47.69	26.20	2.44	0.69	21.03	36.81
S10	Shaanxi	7.90	16.49	22.37	1.36	0.98	24.37	37.30
S11	Hebei	7.98	8.57	8.12	0.68	0.53	24.22	31.96
S12	Henan	8.07	17.79	16.01	1.07	0.75	19.86	35.89
S13	Xinjiang	8.12	19.43	25.25	1.32	0.78	25.49	31.73
S14	Shanxi	8.24	23.17	16.80	1.13	0.95	23.70	37.97
S15	Tianjin	8.29	22.02	24.67	1.42	0.92	24.63	34.18
S16	Gansu	8.37	19.27	11.23	1.05	0.74	23.62	35.22
S17	Shandong	8.65	11.84	13.09	0.93	0.97	21.37	34.55

Soil sample locations are listed in the order of increasing pH.

### Experimental Design

#### Bioaccumulation factors for Pb by corn grain from seventeen soils

According to Grade Two Standard for Pb in the Soil Environmental Quality Standards of China (GB15618-1995), the tested levels of Pb added in soils were shown in Table 2. To obtain the tested levels, a certain amount of exogenous Pb i.e. Pb(NO_3_)_2_ was added to the 8 kg air-dried soils. All the soils were thoroughly mixed and placed into pots (φ×h, 23 cm×26.5 cm). Then the soils were moistened with deionized water to the 60% of field moisture capacity. Each tested level of Pb was replicated three times. Then the soils containing Pb at different levels were covered with plastic film with six small holes for air ventilation. All the pots were kept for 3 months for Pb aging at a greenhouse at the temperature of 25±3°C during the daytime and 20±3°C at night with a natural light photoperiod. During the period of aging, water contents of each soil sample was maintained by adding deionized water every 3 days. After 3 months’ aging, corn seeds of Zhengdan 958 were sown in each soil containing Pb. During the experimental period, soil moisture content was maintained at approximately 60% of water holding capacity through weighing method and adding deionized water to the pots. The corn with grain were sampled and harvested till the corn maturity.

**Table 2:** Soil Environmental Quality Standards of China (GB15618-1995) and the added contents of exogenous Pb (mg.kg^−1^).

pH	<6.5	6.5∼7.5	>7.5
Grade Two Standard	250	300	350
Low Pb	125	150	175
High Pb	250	300	350

#### Bioaccumulation factors for Pb of eight corn species from two soils

Two soils with typical properties sampled from Jiangxi and Shanxi provinces were selected for this experiment. The tested levels of Pb and exogenous Pb addition were the same as those described in the experiment of bioaccumulation factors for Pb by corn from different soils (Table 2). Three replications were conducted on each tested level of Pb. After three months’ aging of soils, the corn seeds, such as Jingketian 183, Chuandan 30, Liaodan 565, Tunyu 88, Zhongdan 808, and Nongda 84,were surface sterilized with 1% (v/v) NaOCl, rinsed, and soaked in distilled water for 24 h at 33°C in dark. Then these seeds were sown in each soil containing Pb. All these pots were placed in the greenhouse at the temperature of 25±3°C during the daytime and 20±3°C at night with a natural light photoperiod.

### Soil Sampling and Analysis

After three months’ aging, soils in the pots of the above two experiments were sampled, air-dried, passed through a <0.20-mm sieve, and then thoroughly mixed. 0.5 g of the soil sample was weighed and added to digestion tubes containing 9 ml HNO_3_ and 3 ml HF.

The corn grains were harvested and oven-dried at 105°C for 30 minutes and then kept at 70°C until the weight of the grains was stable. 0.5 g of corn grain samples oven-dried were weighed and digested in 6 ml concentrated HNO_3_ (70% w/v) and 3 ml H_2_O_2_ in a CEM Mars X microwave oven (CEM Mars) at a pressure of 3.1 MPa. The Pb concentration in the digestion solution from soil and grain was determined by inductively coupled plasma-mass spectrometry (ICP-MS, Agilent 7500a, Agilent Technologies Co. Ltd., USA).

### Data Analysis

The bioconcentration factor (BCF) was calculated as the ratio of the content of Pb in the corn grain to that in the soil (equation 1) [45]–[46].
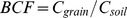
(1)Where *C*_grain_ is the Pb concentration in the corn grain and C_soil_ is Pb concentration in the soil.

SPSS 16.0 for Windows® 107 (SPSS Inc, Chicago, IL, USA) was used for the regression analysis and statistical analysis of the significant differences. Origin 8.0 (OriginLab Co., Northampton, MA, USA) was employed for figure rendering.

### Model

The prediction models for Pb transfer were established through multiple stepwise regression of the Pb BCF from Zhengdan 958 in a wide range of soil properties. The basis of these models is on the basis of equation 2 [38].
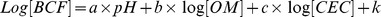
(2)Where Log [BCF], Log [OM], and Log [CEC] were the logarithm base 10 of the BCF values, the content of organic matter (g.kg^−1^) in soils, and the cation exchange capacity (cmol.kg^−1^) of soils, respectively? The slope of soil property parameters such as a, b, and c indicate the impacts of soil properties on Pb accumulation in corn grain. The intercept *k* is the intrinsic sensitivity that characterizes the ability of corn species to absorbing Pb.

### Cross-species Extrapolation

In the process of cross-species extrapolation, an interim alternative was to assume that interactions between Pb accumulation in corn grain and soil pH, OM, CEC were the same among related corn species. In other words, the models stability constants including a, b, c were assumed the same among related species, and the only difference between related species was assumed to be their intrinsic sensitivity (*k*) [38]. The variation of intrinsic sensitivities within a species among plants reflects residual variation [34]. Under the condition of the squared error with the lowest value between predicted BCF value and the measured BCF value calculated as 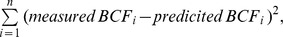 the intercept (*k*) for different models corresponding to various species were obtained through Excel Solver for linear optimization [47].

The accuracy of the model predictions was evaluated by comparing the measured BCF of the other non-model corn species with the predicted BCF from the model. The predictions for other non-model corn species were calculated with the prediction model derived from corn species i.e. Zhengdan 958.

### Analysis of the Reduction of Intra-species Variability

The Pb BCF values of non-model corn species were normalized to the specific soil conditions through the obtained model for Zhengdan 958 and the intra-species variability was computed by equation 3 [47].
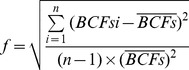
(3)Where the BCF of i-th conditions for specific corn species was normalized to specific soil conditions. *BCFsi* is the value that Pb BCF specific corn species were normalized to i-th soil conditions. 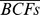 is the mean of n BCFs. *n* is the number of different conditions for specific corn species, *f* is the intra-species variability.

The BCFs that Pb for different corn species was normalized by the prediction models to a set soil condition should be equal. Therefore, the decreases in intra-species variability indicate that normalization processing eliminated soil properties to a certain extent.

## Results

### Major Factors Affecting Pb Accumulation in Corn Grain in Different Soils

**Figure 1:**
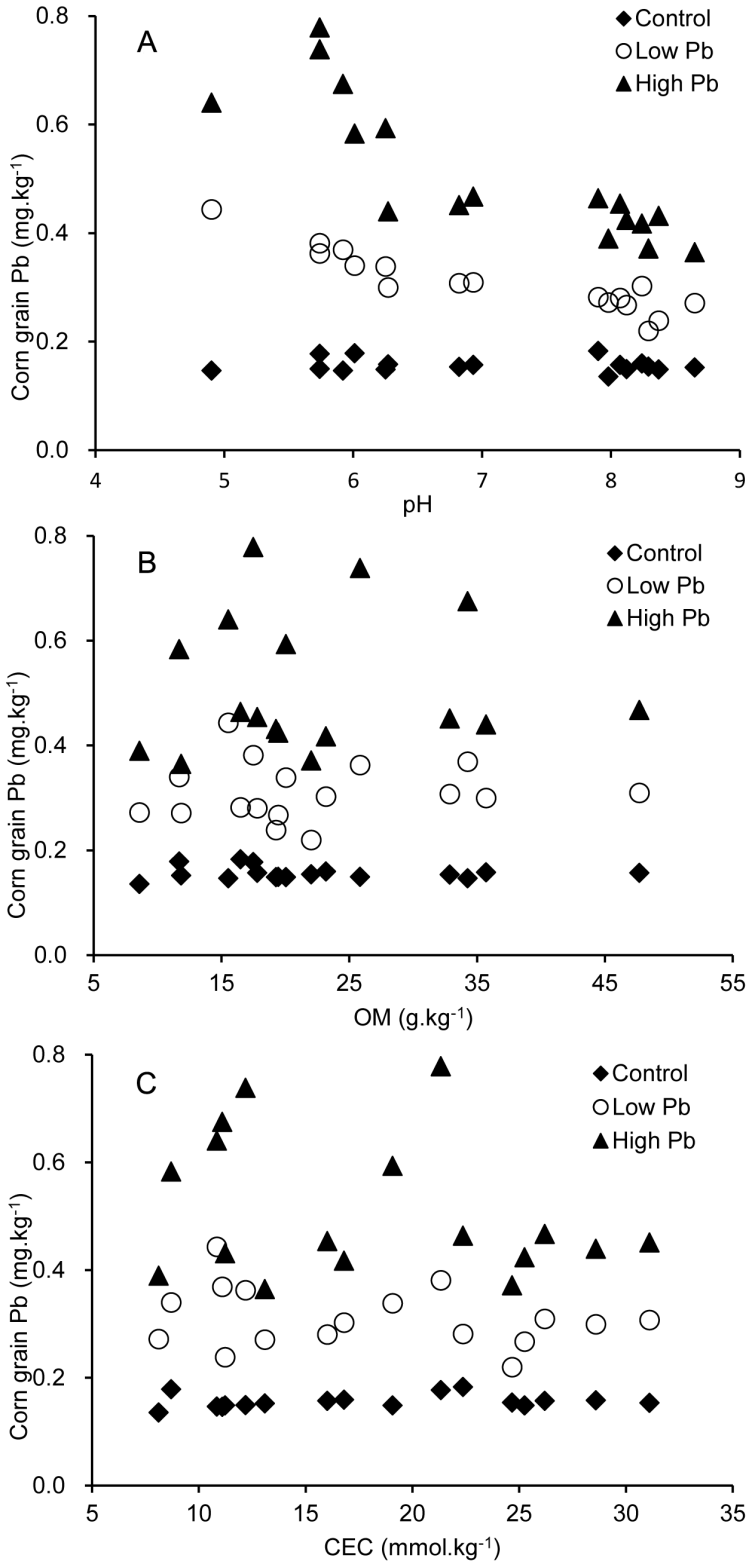
Relationships between Pb in corn grain and soil properties.

The relationships between Pb concentration in corn grain at different Pb levels and soil pH, OM and CEC were shown in Figure 1. At the treatment of low concentration Pb, the Pb concentration in corn grain decreased with the increasing pH of soil. The similar trend was also found at the treatment of high concentration Pb. There was no significant difference was found among Pb concentration in corn grain with the change of pH at the treatment of control. The significantly negatively direct correlations were observed between Pb accumulation in corn grain and soil pH at low concentration Pb, and high concentration Pb, respectively. However, no significantly direct correlations between Pb concentration in corn grain and soil OM, CEC were found.

### Effects of Different Soil Types on Pb Transfer from Soil to Corn Grain

**Figure 2:**
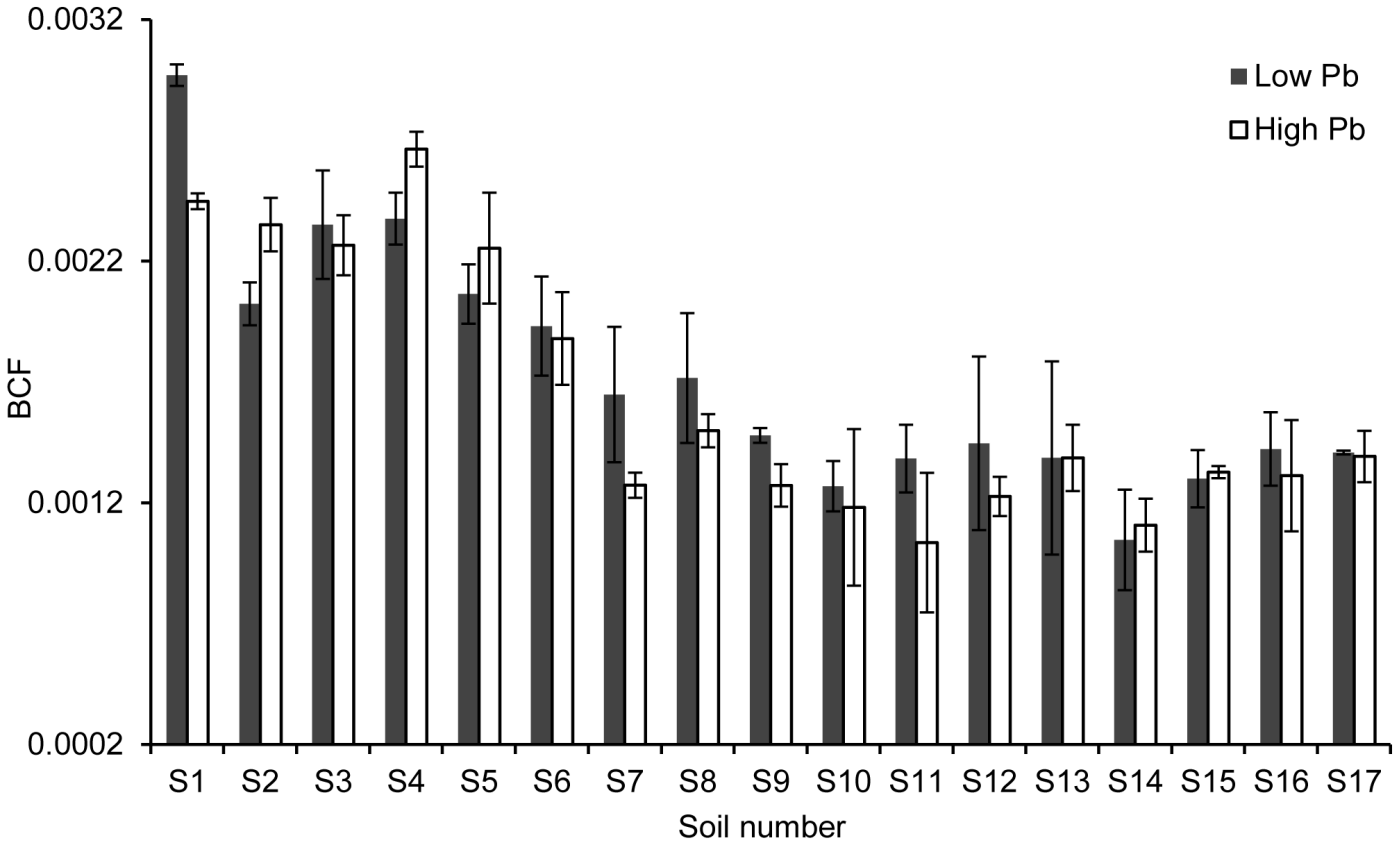
Effects of different soil types on the bioconcentration factor under stress of exogenous Pb.

As shown in Figure 2, the BCF values from Zhengdan 958 under stresses of exogenous Pb significantly differed in 17 soils with a wide range of soil properties in China. The variation tendency of BCF values from Zhengdan 958 was generally consistent at different Pb levels of treatments. These BCF values decreased with the increases of soil pH. The BCF values detected from S1 to S9 (pH from 4.90 to 6.93) were significantly higher than those from S10 to S17 (pH from 7.90 to 8.80). At the treatment of low Pb, the maximum BCF (0.0030) among the 17 soils was observed in S1, and the minimum value for BCF (0.0010) was found in S14. The maximum value was 3.0 folds the minimum. At the same time, the highest BCF (0.0024) values were once again found in S1, and the lowest BCF (0.0010) was found in S11 at the treatment of high Pb, The maximum was 2.40 folds the minimum. These results indicate that Pb under acidic soil conditions is more highly bioavailable and leads to increased absorption by the plants.

### Prediction Models

The prediction models established by stepwise regression were shown in Table 3. The result showed that pH and OM were the major factors affecting Pb accumulation in corn grain. A significantly negative correlation was observed between the Log[BCF] and soil pH and Log[OM] at the different treatments including low Pb, and high Pb, with the R^2^ values of 0.8227, and 0.8955 (*P*<0.001), respectively. There was no significant difference between the two equations at the different treatment of Pb levels.

**Table 3:** Prediction models for the different Pb levels.

Model No.	Lead levels	Prediction models	R^2^	*P*
Model 1	Low Pb	Log[BCF] = −0.098 pH-0.150 log[OM] −1. 894	0.9085	<0.001
Model 2	High Pb	Log[BCF] = −0.108 pH-0.178 log[OM] −1. 806	0.8603	<0.001

### Cross-species Extrapolation

The intercept (*k*) can indicate the sensitivity of corn species to Pb accumulation. The intercept (*k*) for different corn species deduced by two different models were shown in Table 4. In terms of the same corn cultivar, no significant differences were observed among the *k* values for all tested non-model corn species deduced by the same model including model 1, and 2. However, the variation of *k* values among corn species deduced by the model 2 was stronger than that by the model 1. For example, the *k* values of corn species were different with the range from −1.888 to −1.736 deduced by model 1 and with range from −2.295 to −1.823 deduced by the model 2.

**Table 4:** Intrinsic sensitivity (*k*) for non-model species fitted by models from Zhengdan 958.

	Intrinsic sensitivities (*k*)					
PredictionModel	Jingketian 183	Chuandan 30	Liaodan 565	Tunyu 88	Zhongdan 808	Nongda 84
Model 1	−1.823	−1.881	−1.924	−2.295	−2.288	−2.152
Model 2	−1.681	−1.785	−1.846	−1.857	−1.794	−1.717

**Figure 3:**
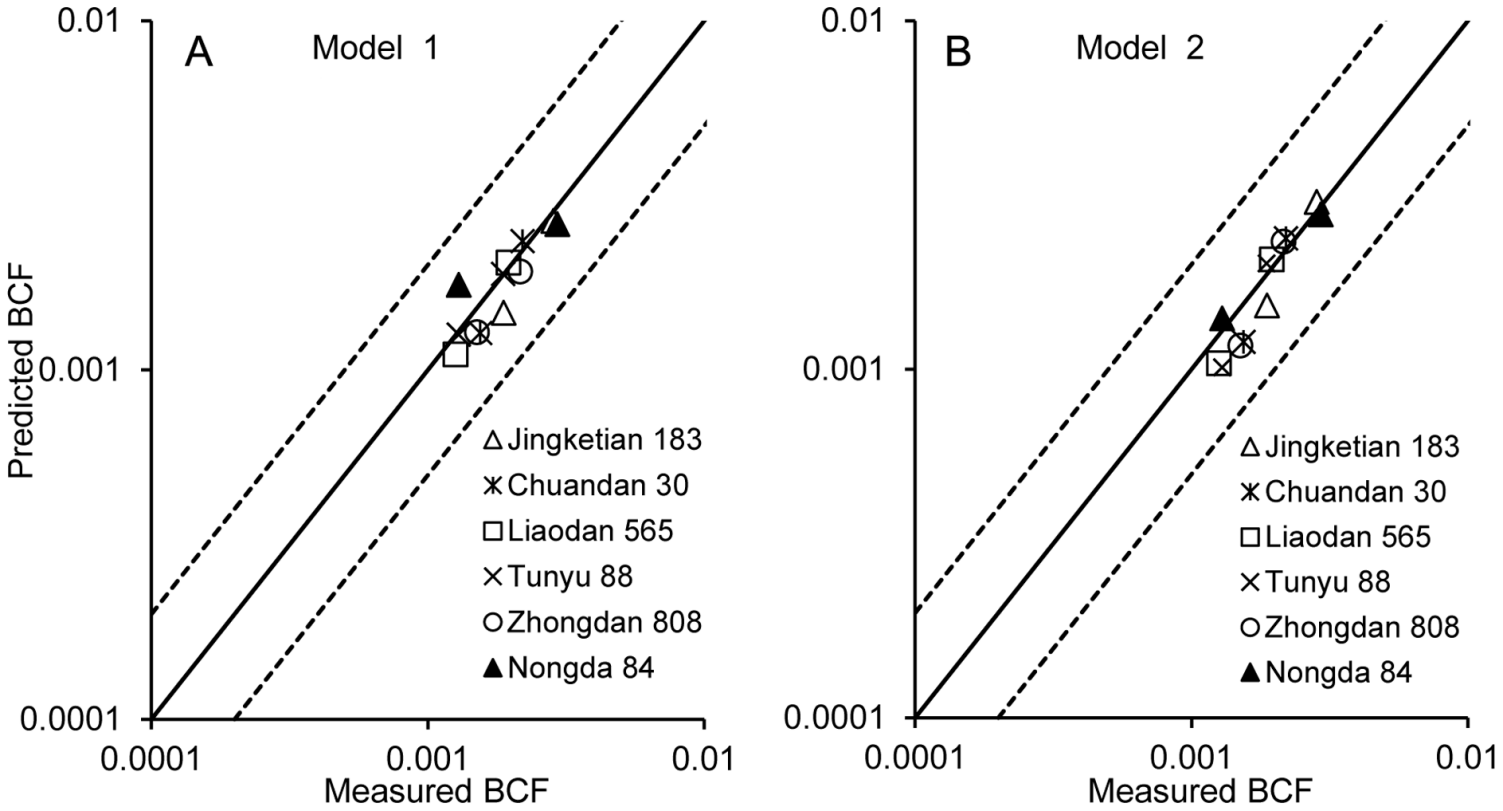
Relativity between measured and predicted BCF values for Pb in non-model corn grains. The predicted BCF values were estimated by model 1 and model 2 in Table 3; the solid line represents a 1∶1 relationship; the interval between two dashed lines indicates a 2-fold prediction interval between the predicted and measured values.

On the basis of the *k* values, the BCF values for non-model corn species were predicted by different models developed for Zhengdan958. The relationship between the predicted BCF values and the measured BCF values for non-model corn species was shown in Figure 3. The ratio between the predicted BCF values and the measured BCF values was within 2-fold interval and close to the solid line of 1∶1 relationship. It indicates that the two models from Zhengdan 958 can be applied to predict the Pb BCF for non-model corn species.

### Reduction of Intra-species Variability

**Figure 4:**
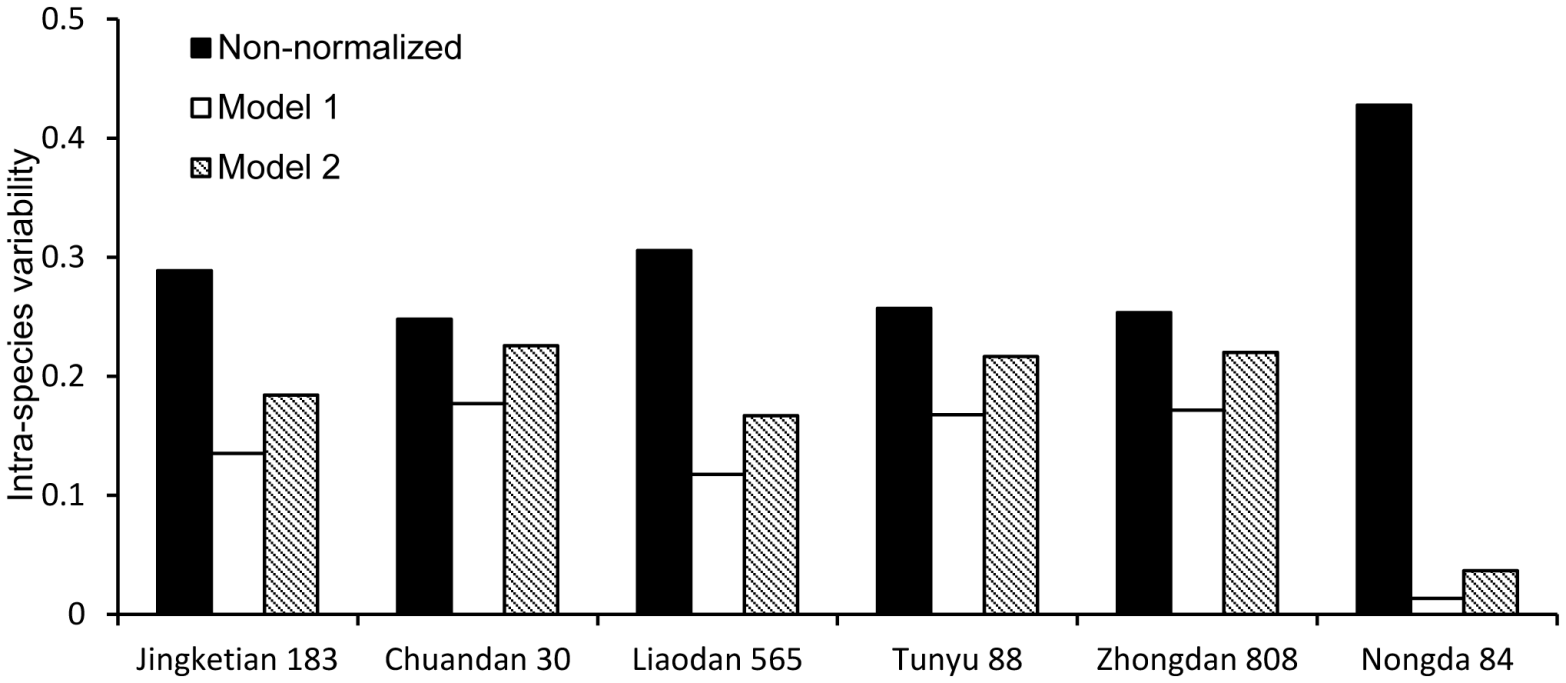
Intra-species variability of Pb BCF. The data from all non-model corn species were normalized with the models listed in Table 3.

Figure 4 shows the intra-species variability of Pb BCF for non-model species which were normalized with the prediction models listed in Table 3. The two prediction models can reduce the measured BCF intra-species variability for all non-model corn species. The intra-species variability of Pb BCF values fitted by model 1 was found to be significantly lower than those by model 1. It indicates that model 1 is more effective in reducing the uncertainty caused by the soil property differences than model 2. This novel result also indicate that model 1 can be more reasonable and to be applied to other non-model corn species to establish the soil Pb SSD curves and to provide an ecological benchmark in China in the future.

## Discussion

The bioavailable concentration of heavy metals is a key factor affecting its uptake and accumulation in plants [48]. In the present study, the results showed that corn grain taken up Pb from soil was mainly governed by soil pH and OM content. The Pb accumulation in the corn grain increased with the decreasing soil pH. This mainly attributed to pH played an important role in Pb uptake by plants from soil, this process through diverse reactions such as adsorption, ionic exchange, redox reactions, and precipitation-dissolution. These reactions are mainly affected by soil pH because of pH affecting Pb speciation and the charge of soil surface groups [49]–[50]. Christensen [51] and Naidu R et al [52] have found that the pH also influence on the solution activity of Pb and the distribution of Pb between the soil phase and the solution phase. The increased sorption of Pb at high pH values could reduce the Pb speciation in soils. In the present study, the BCF values observed in acidic soils (S1 to S9) with low pH values were significantly higher than those in alkaline soils (S10 to S17) with high pH values (Figure 2). These new results suggest that Pb is much more bioavailable under acidic conditions and is easily accumulated in corn grain. In the present paper, it is newly found that the Pb accumulation in corn grain was negatively related with OM. This is alike to the Cu accumulation in plant shoots [53], however it is different from Cd accumulation in corn grain [17]. This may be attributed to organic matter action as a primary sorbent of Pb because of its high sorption affinity [54]–[56].

In the present paper, no significant differences were observed between model 1 and model 2 at the different exogenous Pb concentration. In addition, there were also no significantly differences among Pb BCF values of corn grain at different exogenous Pb concentration, either. This was consistent with the findings of Rezvani and Zaefarian [57].

More importantly, we confirmed that the Pb BCF prediction models from Zhengdan 958 could be used to predict Pb BCF of other non-model corn species according to the soils pH and OM through cross-species extrapolation approach. Further, it was more accurate to apply model 2 to predict the BCF values of other corn species than model 1. Although many prediction models were developed to describe the relationship between plants uptake heavy metals and soil properties in terrestrial ecosystems, these models can’t be extrapolated to non-model plant species, being only for single species [58]–[59]. Only a few studies were carried out to develop the prediction models for one species in aquatic ecosystems and then they are applied to other non-model species [36], [38]. Few prediction models were developed for Pb accumulation in corn grain [60].

**Figure 5:**
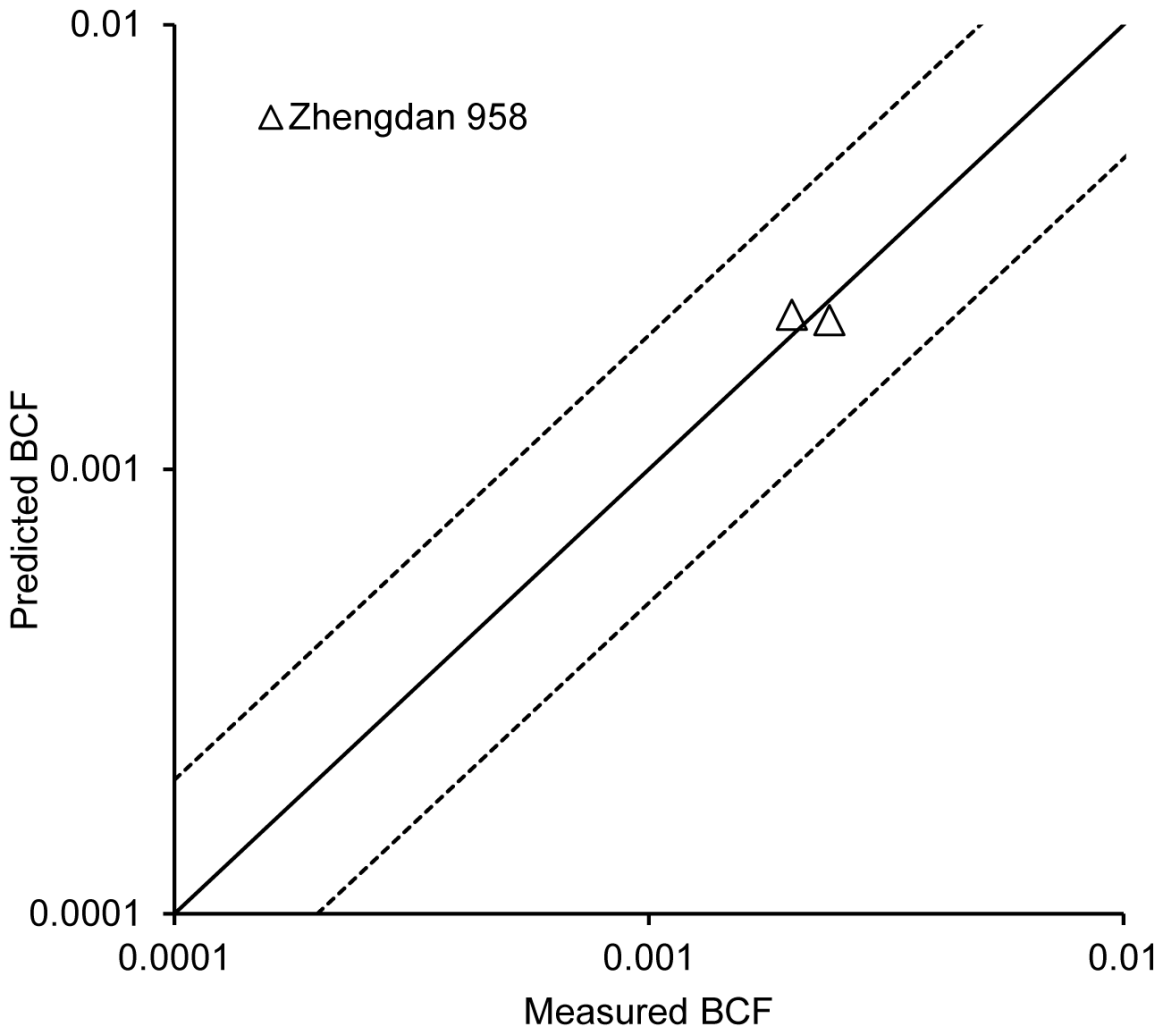
Relativity between measured and predicted BCF values for Pb in corn grains. The measured BCF were obtained from references. The predicted BCF values in Figure 5 were estimated by model 1. The solid line represents a 1∶1 relationship; the dashed lines indicate a 2-fold prediction interval between the predicted and measured values.

In order to further validate the feasibility of the prediction modes developed in the present study, the measured Pb BCF values of corn grains from soils were selected from the references [61], [62], which were shown in Table 5. Although the corn species was also the same as that used to develop the model 1, the properties of the soils used in these references including OM, pH, and so on were different from those in the present paper. Therefore it is suitable that these data were used to test the prediction accuracy of model 1. The relativity between measured and predicted BCF values for Pb is shown in Figure 5. The result showed that the ratios between the predicted BCF values and the measured BCF values were also within 2 folds interval and close to 1∶1 relationship. This indicates that the model 1 developed from Zhengdan 958 in the present study is feasible. It could be applied to other non-model corn species.

**Table 5:** Soil properties and BCF values for Pb from reference.

Cultivation soil	cultivar species	pH	OM (g.kg-1)	CEC (cmol.kg^−1^)	Pb (g.kg^−1^)	BCF	Reference
Soil	Zhengdan 958	6.3	17.6	22.6	30.62	0.0020	[61]
Soil	Zhengdan 958	5.9	38.6	–	30.72	0.0024	[62]

## Conclusions

The soil pH and OM were the most major factors that can control Pb uptake in corn grain. The Pb accumulation in corn grain was negatively related with pH and Log [OM], respectively. The Pb BCF in corn grain can be well predicted by the model 1, which could be applied to other non-model corn species.
